# POSITIVE EVENT DIVERSITY AND AGE: TESTING CURVILINEAR ASSOCIATIONS

**DOI:** 10.1093/geroni/igad104.0926

**Published:** 2023-12-21

**Authors:** Patrick Klaiber, Patrick Hill, David Almeida, Anita DeLongis, Nancy Sin

**Affiliations:** Tilburg University, Tilburg, Noord-Brabant, Netherlands; Washington University in St. Louis, St. Louis, Missouri, United States; The Pennsylvania State University, State College, Pennsylvania, United States; University of British Columbia, Vancouver, British Columbia, Canada; University of British Columbia, Vancouver, British Columbia, Canada

## Abstract

Prior research has found that older adults report more frequent positive events compared to younger and middle-aged adults. It remains unknown, however, to what extent age is related to experiencing positive events spread across different positive event types (i.e., positive event diversity, PED). We therefore examined age differences in PED and its affective well-being correlates across three large daily diary datasets: wave 2 (N = 1919) and the Refresher sample (N = 744) of the National Studies of Daily Experiences, and the Coping with the COVID-19 Outbreak Study (N = 1392). We used Shannon’s Entropy to quantify PED. There was a curvilinear association between PED and age, indicating that PED was higher among middle-aged compared to younger and older adults. On a zero-order basis, PED was correlated with higher positive affect and uncorrelated with negative affect. However, the pattern of results changed when considering positive event frequency as a moderator. Among people with high positive event frequency, PED was linked to higher negative and lower positive affect, but not associated with either positive or negative affect at low levels of positive event frequency. These relationships did not differ by age. This study suggests that middle-aged adults experience the widest range of positive event types, perhaps reflecting the diverse set of social roles that middle-aged adults often fulfill (e.g., worker, parent, partner). In addition, there might be negative consequences to experiencing a lot of positive events across very different contexts which may indicate social role strain.

